# The Microbial Communities of Leaves and Roots Associated with Turtle Grass (*Thalassia testudinum*) and Manatee Grass (*Syringodium filliforme*) are Distinct from Seawater and Sediment Communities, but Are Similar between Species and Sampling Sites

**DOI:** 10.3390/microorganisms7010004

**Published:** 2018-12-26

**Authors:** Kelly Ugarelli, Peeter Laas, Ulrich Stingl

**Affiliations:** Ft. Lauderdale Research and Education Center, Department of Microbiology and Cell Science, UF/IFAS, University of Florida, Davie, FL 33314, USA; kugarelli@ufl.edu (K.U.); peeter.laas@ufl.edu (P.L.)

**Keywords:** seagrass, phyllosphere, rhizosphere, microbiome

## Abstract

Seagrasses are vital members of coastal systems, which provide several important ecosystem services such as improvement of water quality, shoreline protection, and serving as shelter, food, and nursery to many species, including economically important fish. They also act as a major carbon sink and supply copious amounts of oxygen to the ocean. A decline in seagrasses has been observed worldwide, partly due to climate change, direct and indirect human activities, diseases, and increased sulfide concentrations in the coastal porewaters. Several studies have shown a symbiotic relationship between seagrasses and their microbiome. For instance, the sulfur, nitrogen, and carbon cycles are important biochemical pathways that seem to be linked between the plant and its microbiome. The microbiome presumably also plays a key role in the health of the plant, for example in oxidizing phyto-toxic sulfide into non-toxic sulfate, or by providing protection for seagrasses from pathogens. Two of the most abundant seagrasses in Florida include *Thalassia* *testudinum* (turtle grass) and *Syringodium filliforme* (manatee grass), yet there is little data on the composition of the microbiome of these two genera. In this study, the microbial composition of the phyllosphere and rhizosphere of *Thalassia testudinum* and *Syringodium filiforme* were compared to water and sediment controls using amplicon sequencing of the V4 region of the 16S rRNA gene. The microbial composition of the leaves, roots, seawater, and sediment differ from one another, but are similar between the two species of seagrasses.

## 1. Introduction

Seagrasses are found nearly worldwide, excluding Antarctica [1,2], and the total area of seagrass coverage has been estimated between 300,000 to 600,000 km^2^ [3,4]. Seagrasses provide many essential ecological benefits, such as storage of blue carbon, supplying food, shelter, nursery, and ecosystem engineering (reviewed by Ugarelli et al. 2017 [5]). Not only are seagrasses a major carbon sink [6], but also, as a byproduct of photosynthesis, one square meter of seagrass-meadow can emit up to 10 L of oxygen daily [7]. Seagrasses, along with mangroves and coral reefs, also help buffer harsh waves and prevent the stir-up of sediment [8,9]. Their roots extend vertically and horizontally, and the rhizomes extend horizontally, which allows them to withstand the forces of strong tides [8] and prevent uprooting, even with heavily grazed canopies [9]. Seagrass beds also significantly reduce the number of pathogens present in the water column [10].

Many species of fauna depend on seagrasses for food and nutrition. For example, manatees can eat between 30 to 55 kg of seagrass daily [11,12], and sea turtles can eat about 2 kg of seagrass per day [7]. Crustaceans and snails will also occasionally consume seagrass leaves [7,13,14]. Other species that take advantage of seagrasses for food include carnivores who feed on the animals that are frequently found living within the seagrass meadows [13,14,15,16]. Decaying seagrasses serve as food for other organisms such as worms, sea cucumbers, crabs and filter feeders, as well as fauna both near shore and in the deeper ocean where dead seagrass and debris are carried by waves [7]. Decomposition of the seagrasses eventually leads to the release of nutrients, including nitrogen and phosphorous, which can then be reabsorbed by live seagrasses and phytoplankton [13].

Losses in the seagrass population have been observed throughout the world. Waycott et al. 2009 [17] estimated that about a third of the area covered by seagrasses has disappeared since 1879, and so have the ecosystem services provided by them. Siltation [18,19,20], damming and waste disposal [14,21,22], as well as climate change [23], have caused major impacts on seagrass beds. Increases in nutrient concentrations, namely nitrogen and phosphorous [24], due to effluence of fertilizers lead to extensive growth of epiphytes and pelagic algae in the water that block sunlight from reaching seagrass leaves [24,25,26,27,28,29]. Sulfide, a highly toxic compound for animals and plants, is extremely detrimental to seagrasses worldwide [30,31]. Toxic sulfide can be taken up into the shoots because of low oxygen levels in the sediment [32,33]. Normally, seagrasses can detoxify low levels of sulfide by oxygenating their roots [34,35] and through incorporating sulfur into their biomass as thiols [36], but higher concentrations are toxic.

Symbiotic microbes are important for the fitness, growth and survival of plants [37]. Important biochemical processes linked between the host and symbionts include reactions in the carbon, nitrogen, and sulfur cycles. Dissolved organic carbon (DOC) that is exuded from the roots and leaves of the seagrasses can serve as the main carbon source for bacteria associated with seagrasses [38,39,40,41], which includes bacteria that can provide nitrogen to the plant host [42,43,44,45,46]. Biochemical processes in the nitrogen cycle, e.g., nitrification, denitrification and ammonification, occur at higher rates in the rhizosphere of seagrasses than in bare sediments, likely due to microbial activity [42,47,48]. Nitrogen-fixing prokaryotes found both in the phyllosphere [49] and the rhizosphere can provide between 30% and 100% of the nitrogen requirement of seagrasses [42,43,44,45,46]. Sulfate-reduction is higher in the rhizosphere sediment than in non-vegetated sediments [50,51,52]. Even though they prefer anoxic conditions, root surface-associated sulfate reducers seem to have a high tolerance for oxygen [53]. Recently, also sulfur-oxidizing cable bacteria have been reported to be part of the seagrass rhizosphere [54].

The recent and on-going decrease in seagrass beds calls for further research on different aspects of these plants. Microbial community composition, such as the composition of the human gut microbiome, are generally useful bio-indicators for changes that may cause stress on the host. Rather than only relying on the physiological conditions of the plants, analysis of healthy plants and their microbiomes will determine a core-microbiome that can be used as a bio-indicator. Several studies that have explored the microbiome of seagrasses have focused mainly on the genus *Zostera*, but few studies have analyzed the microbial communities associated with *T. testudinum* and even fewer those of *S. filiforme* (reviewed in Ugarelli et al. 2017 [5]) despite their importance and their value to marine animals [55,56,57,58]. In this study, we identified the microbial community composition of different plant sample-types (phyllosphere and rhizosphere) in comparison to seawater and sediment controls of the seagrass species, *T. testudinum* and *S. filiforme*.

## 2. Materials and Methods

**Figure 1:**
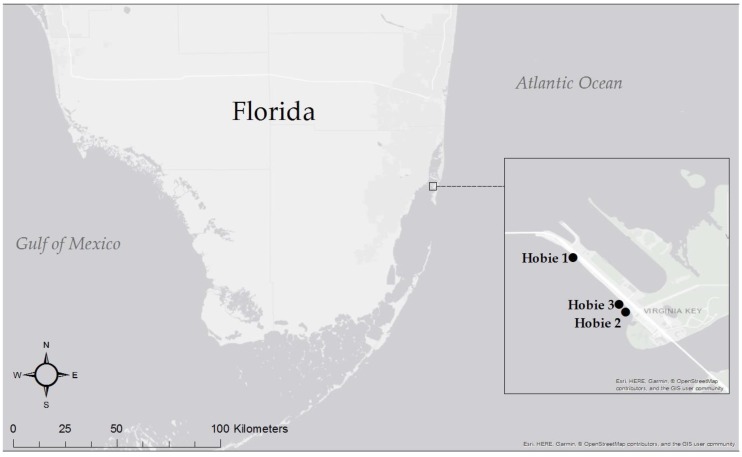
Map of the study area showing sampling locations, Hobie 1 (H1), Hobie 2 (H2), Hobie 3 (H3).

Samples of *T. testudinum* and *S. filiforme* were collected on 14 November 2017, from three sites, Hobie 1, Hobie 2 and Hobie 3, about 0.1 to 1 km apart off the shore of Hobie Island Beach Park near Key Biscayne, Florida, USA (Figure 1). The physico-chemical properties of the seawater at each sampling site were relatively similar (Table 1). Using a spade, about one 0.09 m^2^ portion of the seagrass bed including its sediment was carved out and placed in a bucket. Sediment samples were scooped from the collected seagrass carvings with a 50 mL conical tube, about 5 cm in depth. The remaining seagrass was placed in a 19 L ziplock bag. We collected one 0.09 m^2^ sample of each species per site and one sample of sediment from each seagrass carving. Sediment controls were collected from unvegetated areas about 2 m away from the seagrass bed with 50 mL conical tubes pressed down into the sediment, taking cores of about 5 cm. Using sterile 5 L carboys, seawater was collected from above the seagrass meadows at each site. Due to the proximity of both seagrass species throughout the vegetation, only one sample of water was taken from each site. All seagrass and sediment samples were placed on ice for transfer to the lab. All samples were stored at 4 °C until processing the next day. We refer to leaves, roots, rhizomes, seawater, and sediments as sample-types.

To process the seagrass samples, protocols from Weidner et al. 1996 [59] and White et al. 2015 [60] were adapted, where the microbial community associated with the plants are removed in a series of sonications. Briefly, we gently rinsed the seagrasses with autoclaved seawater to remove loose sediment. The leaves and roots of both species and the rhizome of *T. testudinum* were then detached using sterile blades. For the phyllosphere, 8 cm long segments were cut from the center of the leaves. For analyzing ‘rhizosphere’, the roots were severed from the rhizome and shoots. *T. testudinum* rhizomes were cut to 8 cm in length. About 10 cuttings of leaves, 30 cuttings of roots, and one cutting of the rhizome were placed into separate 50 mL conical tubes containing 30 mL of sterile seawater until the tubes were lightly packed. The tubes were then positioned in a styrofoam raft and placed in a sonication bath filled with deionized water. The samples were sonicated for 12 s using the ME 4.6 ultrasonic cleaner (Mettler Electronics, Anaheim, CA, USA). Using sterile forceps, the samples were transferred to a new conical tube containing 30 mL of seawater and sonicated a second time for 12 s. The third and final sonication ran for 1 min. Because of the smaller rhizome biomass of *S. filiforme* when compared to *T. testudinum*, *S. filiforme* rhizomes were excluded from this study.

The leaves, roots and rhizomes were removed using sterile forceps, and the supernatant was pre-filtered through 100 µm nylon mesh filters (Fisher Scientific, Pittsburgh, PA, USA) to remove the excess plant material. Thereafter, the supernatant was vacuum-filtered through a 5 µm nitrocellulose (MF-Millipore, Darmstadt, Germany) membrane to remove the remaining debris. The bacterial cells were then filtered onto a 0.22 µm nitrocellulose membrane (MF-Millipore, Darmstadt, Germany). Two liters of each seawater sample were also vacuum-filtered through 5 and 0.22 µm filters. All 0.22 µm filters were processed following the manufacturer’s recommended protocol for the Qiagen PowerWater Kit for DNA extractions. DNA from 0.3 g of sediment from the surface of the each conical tube was extracted using the Qiagen PowerSoil kit for DNA extraction. All DNA extracts were kept at −20 °C until preparation for sequencing.

DNA sequence data was generated using Illumina paired-end sequencing (151 bp × 12bp × 151 bp MiSeq run) at the Environmental Sample Preparation and Sequencing Facility at Argonne National Laboratory (Chicago, IL, USA). DNA extracts were used as templates for the amplification of the V4 hypervariable region of the 16S rRNA gene [61]. The primer pair used was 515F-806R, which contained adapter sequences as well as a twelve-base barcode sequence for multiplexing in the reverse amplification primer. Each 25 µL PCR reaction contained 9.5 µL of MO BIO PCR Water (Qiagen, Germantown, MD, USA), 12.5 µL of QuantaBio’s AccuStart II PCR ToughMix (Quantabio, Beverly, MA, USA; 2× concentration, 1× final), 1 µL Forward Primer (5 µM concentration, 200 pM final), 1 µL Golay barcode tagged Reverse Primer (5 µM concentration, 200 pM final), and 1 µL of template DNA. The conditions for PCR were as follows: 94 °C for 3 min to denature the DNA, with 35 cycles at 94 °C for 45 s, 50 °C for 60 s, and 72 °C for 90 s, and a final extension of 10 min at 72 °C to ensure complete amplification. Amplicons were then quantified using PicoGreen (Invitrogen, Eugene, OR, USA) and a plate reader (Infinite^®^ 200 PRO, Tecan, Männedorf, Switzerland). Once quantified, volumes of each of the products were pooled into a single tube to represent each amplicon in equimolar amounts. This pool was then cleaned up using AMPure XP Beads (Beckman Coulter, Indianapolis, IN, USA), and then quantified using a fluorometer (Qubit, Invitrogen, Eugene, OR, USA). After quantification, the molarity of the pool was determined and diluted down to 2 nM, denatured, and then diluted to a final concentration of 6.75 pM with a 10% PhiX spike for sequencing on an Illumina MiSeq. The raw data has been submitted to the BioProject database and is available at: http://www.ncbi.nlm.nih.gov/bioproject/511001.

The QIIME 2 microbiome analysis package [62], the successor to QIIME [63], was used for sequence analysis. Therein, quality filtering, chimera identification and merging of paired-end reads was carried out with the DADA2 plugin [64]. SILVA release 132 (Ref NR 99) taxonomy [65] and q2-feature-classifier [66] were used for classification of the 16S rRNA gene sequences. Data filtering and statistical analysis were completed through R version 3.2.0 [67]. Sequence variants (SVs) classified as chloroplasts or mitochondria were discarded from the dataset. Several additional R packages were used for data analysis and visualization: SeqinR [68], clusterSim [69], reshape2 [70], ggplot2 [71], VennDiagram [72], and vegan [73]. Analyses of predictive metagenomes were done with the MicrobiomeAnalyst Shotgun Data Profiling (SDP) module [74], which determines functional diversity profiles of the KEGG Orthology (KO)-annotated genes. ArcMap™ from the ArcGIS^®^ software by Esri (Redlands, CA, USA), version 10.5.1 [75] was used to make the map in Figure 1.

## 3. Results

A total number of 2,128,087 sequences that comprised 17,686 sequence variants (SVs) were generated in this study (Table 2). The number of SVs in the turtle grass rhizosphere was 3373, 2771 SVs were recovered from the manatee grass rhizosphere, and 3597 SVs were present attached to the turtle grass rhizome. The turtle grass phyllosphere had 3347 SVs and the manatee grass phyllosphere had 4062 SVs. Both of the seagrass sediments contained 4033 SVs, and the sediment control had 4650 SVs. The water samples contained the least amount of SVs with only 757.

### 3.1. Alpha Diversity

After normalization, the alpha diversity of the bacterial communities was generally higher in the phyllosphere, rhizosphere and sediment than in the seawater samples (Figures S1 and S2, Tables S1 and S2). The phyllosphere and the sediment control communities have the highest species richness, followed by the rhizomes. The root and seagrass sediment communities showed similar alpha diversities. The sampling depth covered most of the diversity within the samples, as the rarefaction curves of most samples plateaued between 1000 to 2000 sequences. The highest diversity was found in the manatee grass leaves from Hobie 2 (Figure S2).

### 3.2. Beta Diversity

**Figure 2:**
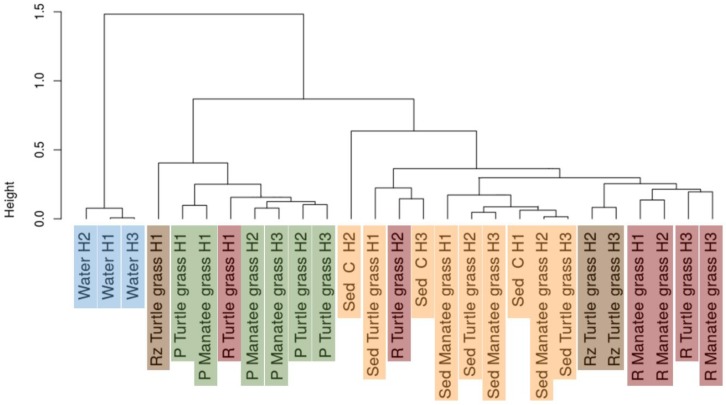
Dendrogram of the weighted Unifrac distance matrix of the microbial communities in each sample of water, sediment, phyllosphere and rhizosphere of both manatee grass and turtle grass, and the rhizomes of turtle grass. (H1: Hobie 1; H2: Hobie 2; H3: Hobie 3; P: phyllosphere; R: rhizosphere; Rz: rhizome; C: control; Sed: sediment). Colors indicate different sample-types.

The community compositions of the different sample-types were clearly distinct (Figure 2): The root and rhizome communities clustered more closely together and were more similar to the sediments than to the water samples. The phyllosphere samples grouped separately from the water and the belowground samples, and the water samples were the most distinct group and clustered separately from all other samples.

### 3.3. Sequence Variants and Abundant Genera

**Figure 3:**
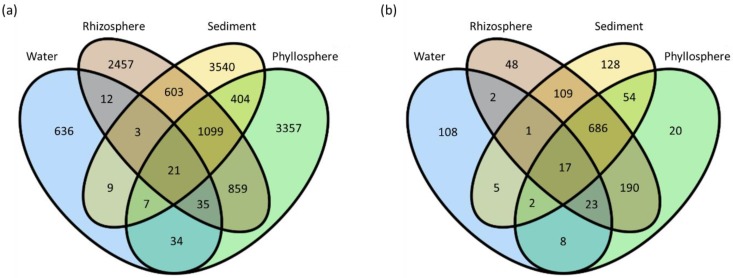
Venn diagrams comparing the number of sequence variants (SVs) shared among the water, rhizosphere, phyllosphere, and seagrass sediments (**a**) Complete set of SVs, (**b**) SVs that make up more than 0.01% of the total SVs.

Due to the large quantities of unique SVs in each sample, only the most abundant SVs that comprised more than 0.01% of the total SVs were considered in order to compare the main members of the microbiome and to exclude rare taxa (or potential sequencing errors) (Figure 3a). Almost half (49%) of the abundant SVs were shared among seagrass-associated sample-types—phyllosphere, sediment, and rhizosphere (Figure 3b). Only about 1.2% of the abundant SVs were common to all the samples including the water.

#### 3.3.1. Aboveground

**Figure 4:**
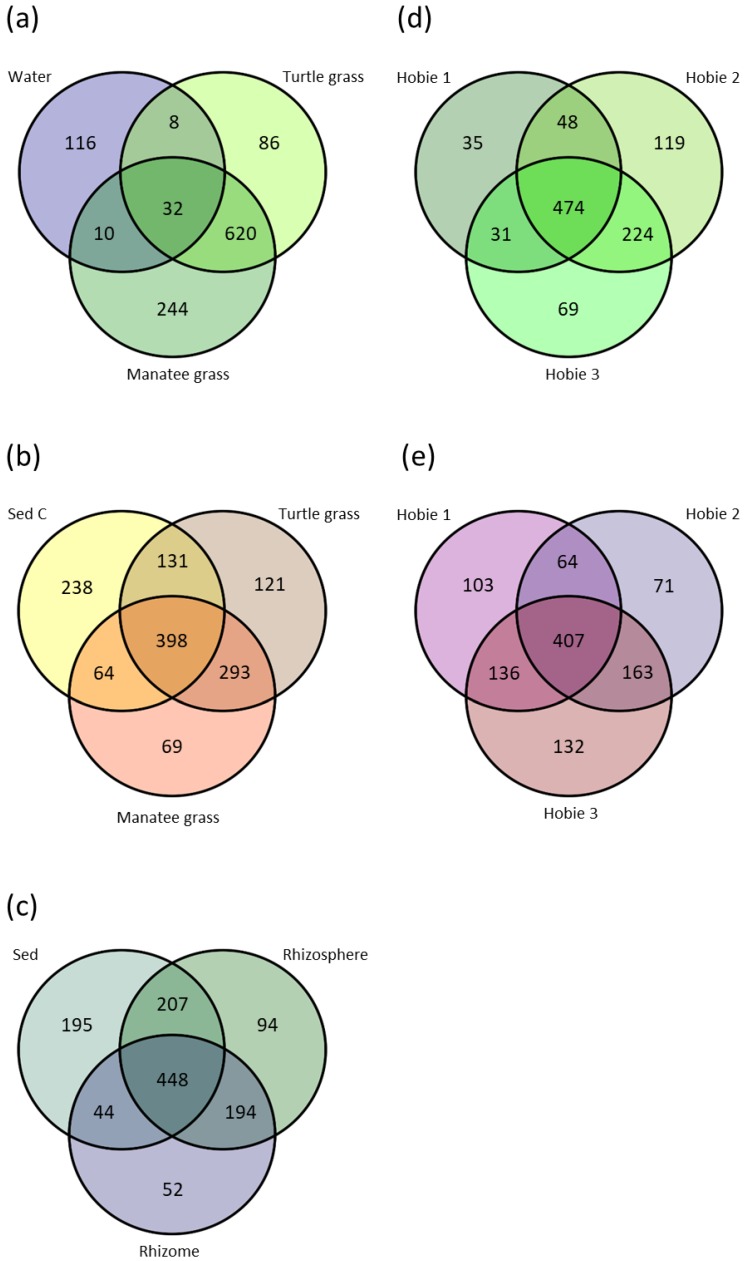
Venn diagrams comparing the most common SVs that comprise more than 0.01% of the total SVs unique to or shared among (**a**) all the phyllosphere and water samples, (**b**) all the rhizosphere and sediment control samples, (**c**) the sediment, rhizospheres, and rhizomes of turtle grass, (**d**) all the phyllospheres of the sample sites, and (**e**) all the rhizosphere of the sample sites. (Sed: sediment; C: control).

Aboveground samples showed a clear difference between the leaf communities of both species and their surrounding seawater (Figure 4a): Phyllospheres of both seagrass species were more similar to each other than they were to the seawater, with 56% of all the SVs found only on the seagrass leaves of both species. Of all the SVs, 3% were common to both phyllospheres and seawater together, and less than 1% of the SVs were shared between the phyllosphere of either seagrass species and the seawater.

**Figure 5:**
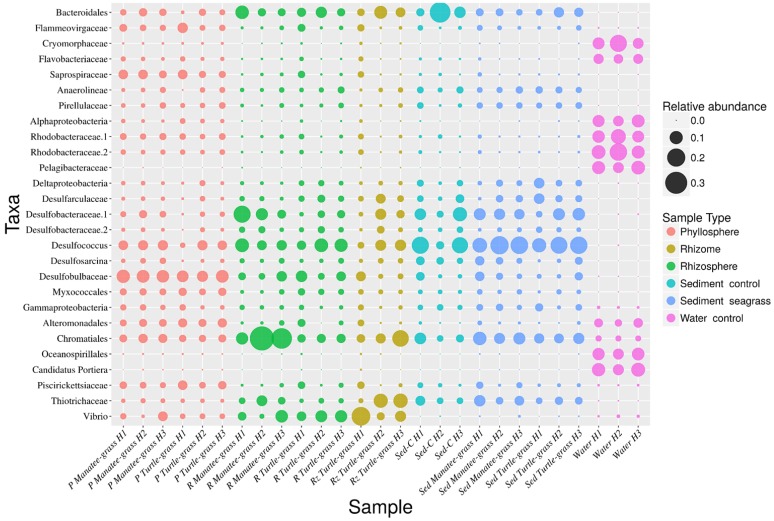
Relative abundance of the taxa comprising more than 1% of the bacterial sequences in each sample. Taxa are classified to the lowest rank possible according the classifier. (H1: Hobie 1; H2: Hobie 2; H3: Hobie 3; P: phyllosphere; R: rhizosphere; Rz: rhizome; C: control; Sed: sediment).

The most abundant genera common to both leaves and seawater were *Alphaproteobacteria*, *Rhodobacteraceae*, and *Alteromonadales* (Figure 5). *Crymorphaceae*, *Flavobacteriaceae*, *Pelagibacteraceae*, *Chromatiales*, *Oceanospirillales*, and *Candidatus* Portiera were abundant mainly in seawater. *Piscirickettsiaceae*, *Myxococcales*, *Desulfobulbaceae*, *Desulfococcus*, *Saprospiraceae*, and *Flammeovirgaceae* were abundant mainly in the phyllospheres.

#### 3.3.2. Belowground

Unlike the aboveground sample-type communities, the belowground communities showed similarity among each other and the surrounding sediments (Figure 4b). The rhizospheres of both species are more similar to each other than to the sediments controls, with 22% of the SVs found only in the two rhizospheres, while they shared less than 10% of the SVs each with the sediment controls. However, 30% of the SVs were present on the roots of both species and the sediment controls.

The turtle grass rhizome and root communities were compared to the turtle grass sediments (Figure 4c). The number of SVs common to only the roots and the sediment was 17%, and the number of SVs common to only the roots and the rhizome was 16%. The rhizome shared 4% of the SVs with only the sediment. In the rhizomes, roots, and sediments of turtle grass, 36% of the belowground SVs were present.

The predominant taxa common to all belowground components were *Chromatiales*, *Desulfococcus*, *Desulfobacteraceae*, and *Bacteroidales* (Figure 5). *Gammaproteobacteria*, *Desulfosarcina*, *Deltaproteobacteria*, *Pirellulaceae*, *Thiotrichaceae*, and *Anaerolineae* were the most prevalent taxa in the sediments while *Vibrio* and *Thiotrichaceae* were more abundant in the rhizome and roots.

#### 3.3.3. Leaves vs. Roots and Rhizomes

We compared the microbial communities of the leaves and roots of both seagrass species to the turtle grass rhizomes, and found that 47% of the SVs were common to these three sample-types (Figure S3). 6% of the SVs were only found on the leaves and 5% were exclusive to the roots. 1% of the SVs were only found on the rhizomes and phyllospheres, and 8% of the SVs were only present on the rhizome and roots.

#### 3.3.4. Turtle Grass Sample-Types

When comparing the leaves, rhizome, roots, and sediments of *T. testudinum*, 28% of the SVs were present in all sample-types (Figure S4g). The turtle grass sediment was more diverse, with over 13% of all the SVs present only in the turtle grass sediment. 6% of the most abundant SVs were only recovered from both the phyllosphere and rhizosphere of turtle grass.

#### 3.3.5. Manatee Grass Sample-Types

In manatee grass, 38% of the abundant SVs were shared between the leaves, roots, and manatee grass sediments (Figure S4h). The phyllosphere and the rhizosphere communities were more similar to each other than to the sediment communities, with 19% of the SVs present only in these two sample-types and not the sediment. Interestingly, the sediment and the phyllosphere alone shared 10% of the SVs, while the sediment and the roots alone shared 5%. The sediment is more distinct than the other sample-types in manatee grass, with more than 16% of the SVs present only in the sediment. 

#### 3.3.6. Differences in Genera-Abundance by Seagrass Species

Several differences between the two seagrass species were observed based on the abundance of the microbes (Figure 5). In the roots of manatee grass, *Desulfobacteraceae* and *Chromatiales* were more abundant, while in turtle grass unclassified *Deltaproteobacteria* and *Vibrio* were more abundant. The sediments of turtle grass contain more *Bacteroidales* and unclassified *Deltaproteobacteria,* whereas the sediments of manatee grass contain more *Desulfobacteraceae* and *Thiotrichaceae*. The phyllospheres were mostly uniform; however, *Desulfobacteraceae* is more prevalent in manatee grass leaves.

#### 3.3.7. Seawater by Site

The seawater samples show great resemblance among their microbial communities, with 75% of the SVs present in all sites (Figure S5). 5% of the SVs are present only in Hobie 2 and Hobie 3, while 2% and 3% are only in Hobie 1 and Hobie 3, and Hobie 1 and Hobie 2, respectively. Hobie 2 seems to be more unique than the other two sites with about 11% of the abundant SVs exclusively found here.

#### 3.3.8. Phyllosphere by Site

The phyllosphere communities of both species in Hobie 2 and Hobie 3 shared 22% of the SVs. Less than 5% of the SVs are found only between Hobie 1 and either Hobie 2 or Hobie 3. Nonetheless, 47% of the SVs were present in all three locations (Figure 4d), suggesting that the leaf communities of all three sites are very similar.

#### 3.3.9. Rhizosphere by Site

The rhizospheres of both species in Hobie 1 are more similar to Hobie 3 than Hobie 2, with about 13% of the SVs present only in Hobie 1 and Hobie 3, and 6% only between Hobie 1 and Hobie 2 (Figure 4e). 15% of the SVs were exclusively found in Hobie 2 and Hobie 3. The root communities of all three sites were similar, with 38% of the SVs present in all the sites.

#### 3.3.10. Differences in Abundance of Taxa by Site

Several of the abundant taxa were present in the same sample-types from each site; however, their relative abundances differed. For example, *Chromatiales* was present in all the samples, but they are especially prevalent in manatee grass roots and even more so in sites Hobie 2 and Hobie 3 (Figure 5). *Vibrio* were mostly abundant in the turtle grass rhizome in site Hobie 1, *Desulfobacteraceae* was mostly prevalent in manatee grass roots from Hobie 1, unidentified *Thiotrichaceae* was especially prevalent in the rhizomes of sites Hobie 2 and Hobie 3, and unidentified *Bacteroidales* was more abundant in the sediment control of site Hobie 2.

Despite the similarities in the seawater properties (Table 1), there were also differences per site in the abundance of the microbes of the seawater communities. *Flammeovirgaceae* is more abundant in the water of Hobie 2 than it is in Hobie 1 or Hobie 3, and *Alphaproteobacteria* and *Pelagibacteraceae* are more abundant in both Hobie 1 and Hobie 3 than in Hobie 2 (Figure 5). The phyllosphere communities seemed to be more uniform in the abundances, but turtle grass in Hobie 1 contained less *Bacteroidales, Anaerolinea, Deltaproteobacteria, Desulfarculaceae, Desulfobacteraceae, Desulfococcus, Desulfosacina, Thiotrichaceae* and *Vibrio* than the other sites for both species. Turtle grass from Hobie 1 did contain more *Piscirickettsiaceae* than the other phyllospheres, and manatee grass from Hobie 3 contained more *Vibrio*.

### 3.4. Predictive Metagenome Analysis

After analyzing the sequencing results through a functional diversity profiling platform, most of the significant genes that were distinct in the different sample types appear to be involved in amino acid metabolism or in transport systems (Table S3). More significant differences were found in the water samples compared to the rest of the sample-types (Table S3; Figure S8). The rhizosphere is predicted to contain more alcohol dehydrogenase and aldehyde oxidoreductase genes, while the sediment communities are predicted to contain more NAD-dependent aldehyde dehydrogenase genes, and the phyllosphere is predicted to contain more thymidylate synthase genes.

## 4. Discussion

### 4.1. Microbial Communities Across Seagrass Sample-Types

Similar studies that have compared the microbiomes of other seagrass species, namely *Zostera marina* and *Zostera japonica* [76] and *Zostera marina, Zostera noltii* and *Cymodocea nodosa* [77], have found that the microbial communities differ from site to site and sample-type to sample-type, but not from species to species when comparing different geographical locations. Our study on the microbiome of the two seagrasses, *T. testudinum* and *S. filiforme*, suggest a similar pattern, revealing clear differences in the microbial communities of each sample-type—leaves, roots, sediment and seawater—and similarities among sampling sites and seagrass species. The communities of the two seagrass species, although very similar, still contained microbes and SVs that were unique to each species. This perhaps is due to the phylogenetic distance of the two seagrass species, where *Thalassia* belongs to the family *Hydrocharitaceae* and *Syringodium* belongs to the family *Cymodoceaceae*, and subsequent differences in morphology and physiology.

The difference in the community composition of the different plant sample-types is likely due to the different organic compounds provided from each. For example, seagrasses are known to support their phyllosphere communities [78,79] by providing a carbon source for them [38,80]. The age of seagrass leaves seems to also influence the phyllosphere, as Törnblom and Søndergaard 1999 [79] show that the bacterial community of the leaves of *Z. marina* increasingly synthesizes proteins as the leaves age, possibly due to excretions from the plant.

The rhizosphere mainly contains microorganisms involved in the biochemical cycles of nitrogen [81], sulfur [52,81,82], and carbon [38,83,84,85] that are supported in part by the root exudates. The microbial diversity in the rhizosphere is also influenced by the mostly anoxic conditions of the sediment, oxic microzones around the roots, and the more oxygenated aboveground portions of the plants [52]. A recent study by Martin et al. 2018 [54] shows that sulfur-oxidizing cable bacteria are present near the roots of *Zostera muelleri* and *Halophila ovalis*, which also leads to a decreased concentration of sulfide near the roots. Nielsen et al. 2001 [81], who studied *Zostera noltii* and *Spartina maritima*, proposed that high sulfate and acetylene reduction rates observed on the rhizomes and roots indicate the importance of these habitats for sulfate-reducing and nitrogen-fixing bacteria. This study also indicated that nitrogen-fixing sulfate-reducers provide a nitrogen source for the rhizosphere community as well as for the plant itself. They further suggested that the energy for these cycles is provided by soluble exudates from the seagrass host, such as sucrose. Using stable carbon-isotope ratios, seagrasses have been shown to provide substantial amounts of carbon for their microbiome in *Posidonia oceanica* and *Cymodocea nodosa* [84], and *T. testudinum* [80,85].

### 4.2. Site and Species Comparison

The microbial communities of seagrasses do not differ only across the plant sample-types [76,77,86,87,88,89,90], but are also distinct from the sediment [77,89,90] and seawater [76,77]. When comparing the phyllosphere, rhizosphere and sediment communities of *T. testudinum* and *S. filiforme*, each species has SVs that can be considered “species-specific” (Figure 4a–b). However, more than 50% of the SVs were common in both species. When comparing the three sampling sites, few SVs were site-specific, while most were common to all (Figure 4d–e, Figure S5). Studies that have found distinct differences in the microbial communities between sampling sites, e.g., Fahimipour et al. 2017 [90], Bengstton et al. 2017 [88] and Cúcio et al. 2016 [77], sampled from locations that were farther apart and more diverse. Likely due to the proximity of our sampling locations, the microbial communities of these two seagrass species were similar from site to site.

### 4.3. Water and Phyllosphere

Interestingly, Fahimipour et al. 2017 [90] found the microbial communities of the leaves and the seawater samples were similar in a world-wide study of *Zostera marina*. In our study, the seawater communities were vastly different from the plant and sediment communities. The alpha diversity found in the water samples was much less than the diversity of the plant and sediment samples (Figure S1). While the microbial community of the seawater was slightly more related to the leaf-microbes (Figure 2), the seawater samples only shared 3% of the most abundant SVs with the phyllospheres of both species (Figure 4a). The differences between this study and Fahimipour et al. 2017 [90] might be explained by differences in the studied seagrass species and sampling locations, or due to variations in sampling methods.

The seawater samples in this study were less diverse than the other sample-types and contained genera that were unique to the seawater. Curiously, one of the abundant genera in the seawater was *Candidatus* Portiera, which is known as an obligate endosymbiont of whiteflies [91]. This candidate genus was also abundant in seawater and brackish water samples in other studies [92,93,94,95], and has been found in oysters [96]. The order this genus belongs to, *Oceanospirillales*, is a common salt-tolerant taxon [97]. The sequence classified in QIIME 2 as *Candidatus* Portiera likely belongs to an undetermined *Oceanospirillales* genus, as a check using NCBI’s BLAST function suggested.

### 4.4. Roots, Sediments, and Rhizomes

The sediment and the rhizospheres of both seagrass species had very similar microbial communities, but were still distinct enough to cluster mostly separately (Figure 4b, Figure 2). The proximity of the rhizospheres and sediments might lead to the observed similarities in their communities. The rhizome communities also resembled the root and sediment communities (Figure 4c), but the rhizome contained SVs that were not present in either the sediment or the roots, indicating that its microbial community is distinct. The rhizome is essentially a stem and serves as a means of asexual reproduction where the seagrass clones spread [98]. Since its function differs from the rest of the plant, it seems probable that the microbial community of the rhizome also differs. More research on the microbial community of the rhizome compared to those inhabiting the roots is needed. Many studies on the microbes associated with the rhizome comprise both roots and rhizome as part of the rhizosphere [86,99,100]. Wahbeh and Mahasneh 1984 [101] compare the amount of bacteria on the rhizome, roots and leaves separately and show that bacterial loads differ among sample-types. Insufficient research has since been done on the bacterial composition of the rhizome compared to those on roots. Several studies have looked at the different microbial biochemical cycles occurring on the rhizome apart from the roots [53,102,103,104]; however, few have analyzed the makeup of the microbial community. Recently, Vann et al. 2017 [87] showed that the community of the rhizome is distinct from the community of the roots and leaves in *Z. marina*. To the best of our knowledge, no studies have investigated the rhizome communities of other seagrass species.

### 4.5. Core Microbiome

Several studies show that the microbial communities of different seagrass species are similar at the same site but vary between different sites and sample-types [76,77]. However, the distinctions in the different sites do not apply to the whole microbial community, and some studies indicate the presence of a core microbiome in seagrasses [76,77,88,89,90]. Our results are consistent with these findings, considering the geographical proximity of our samples (Figure 1) and the similarities between the microbiome in these sites (Figure 4d–e, Figure 5, Figure S5). Due to the overlapping microbial communities of the two seagrass species, we combined the two species when considering the SVs in the core microbiome. The abundance of the microbes differs between species in the same site (Figure 5). For instance, *Chromatiales* is clearly more abundant in the manatee grass samples than it is in the turtle grass samples. Varying ratios might also hold true in the core microbiomes. The relative abundance of certain taxa might be species specific or the result of differing environmental conditions in the different locations of the seagrasses.

The most abundant taxa in our samples were also commonly found in other studies. For instance, Cúcio et al. 2016 [77] found that the most abundant taxa in all three seagrasses (*Z. marina*, *Z. noltii* and *C. nodosa*) were *Gammaproteobacteria*, *Deltaproteobacteria*, *Bacteroidia*, *Epsilonproteobacteria*, *Anaerolineae*, *Acidimicrobiia*, and *Alphaproteobacteria*. In the rhizosphere, the most abundant taxa were *Epsilonproteobacteria* (family *Helicobacteraceae*), *Acidimicrobiia*, *Gammaproteobacteria* (order *Chromatiales*), *Deltaproteobacteria* (genus *Desulfococcus*), and *Clostridia* (order *Clostridiales*). Ettinger et al. 2017 [89] found that *Z. marina* contained *Bacteroidia*, *Clostridia*, *Flavobacteriia*, *Saprospirae*, *Anaerolineae* as well as *Alpha*-, *Delta*-, *Epsilon*- and *Gammaproteobacteria* in its microbiome. Bengtsson et al. 2017 [88] found the abundant sequence variants to be *Proteobacteria*, *Bacteriodetes*, *Cytophagia*, *Cyanobacteria*, *Verrucomicrobia*, *Planctomycetes*, *Plastids*, and *Actinobacteria* in the leaves of *Z. marina*. In our study, we found *Gammaproteobacteria* in all our samples, though they were slightly more abundant in the roots and rhizome, *Deltaproteobacteria* in all the samples except seawater, and *Alphaproteobacteria* and *Flavobacteriia* mostly in seawater and phyllospheres (Figure 5). Table 3 shows a comparison of the abundant taxa in several studies and in our study.

The core microbiome of seagrasses, as the current and cited studies suggest, is likely composed of *Alphaproteobacteria* and *Gammaproteobacteria* (present in all the studies), *Acidimicrobiia*, *Clostridia*, and *Deltaproteobacteria* (present in six out of seven) and *Betaproteobacteria* (present in five out of seven studies) (Table 3). In our study, *Betaproteobacteria* and *Clostridia* were also present, although they were not abundant (data not shown). These classes might represent species-specific taxa that are mostly present in *Z. marina* and not as prevalent in *T. testudinum* or *S. filiforme*. The location of the sampling site might also contribute to the presence or absence of these taxa. *Betaproteobacteria* and *Clostridia* were not considered abundant in some of the other studies despite analyzing the same seagrass species ([89,90], Table 3). Cúcio et al. 2016 [77] suggests that the core microbiome should be present in all the species of seagrasses. It is possible that the core microbiome may not be exactly the same for all seagrasses since environmental conditions play a major part in the abundance of these microbes [86]. In order to not exclude microbes that were outcompeted or overshadowed by other opportunistic ‘non-members’ of the core microbiome under unfavorable conditions, as has been seen in other plants [105], we should study the microbiome of seagrasses that are not under stress when considering the core microbiome.

### 4.6. Predictive Metabolism

The sequences were run through a predictive metagenomics analysis program, revealing that many of the significantly distinct genes in the different sample-types were associated with amino acid metabolism or transporter proteins (Table S3; Figure S8). Only 30 out of more than 6000 genes were predicted to be significantly different in their abundance per sample-type, and most of these were prevalent in the water, which is likely due to its very different microbial community compared to other sample-types’ (Table S3; Figure 2). One of the few genes that were predicted to be more significant in the rhizosphere was alcohol dehydrogenase. As discussed by Cúcio and colleagues [77], bacteria in the class *Deltaproteobacteria*, namely *Desulfobacteracea* and *Desulfobulbacea*, are known to use sulfates or sulfite as electron acceptors to oxidize alcohol into carbon dioxide. In seagrass roots, there is a lack of oxygen during the night cycle, which encourages the plant to ferment, releasing ethanol into the surrounding environment [77,106]. These bacteria likely utilize the ethanol released from fermentation in the rhizosphere at nighttime, and release hydrogen sulfide in the daytime [77,107]. Some *Desulfococcus*, which are known sulfate-reducers, also possess the genes for alcohol dehydrogenase [108]. The abundance of these *Deltaproteobacteria* in our rhizosphere samples (Figure 5) likely explains the predicted significant differential abundance of alcohol dehydrogenase genes in our rhizosphere samples.

### 4.7. Processing Methods Comparison

Sonication has been used successfully to detach epiphytes from macrophytes [59,109]; however, it is not commonly used in recent seagrass studies. More common techniques include directly extracting DNA from the samples [87,89,90], rinsing samples before processing [76,77,86,88], and scraping samples off the leaves with cotton swabs [88]. Most of these methods involve pelleting the cells and then processing the samples. Our methods included sonications and vacuum-filtration. Eva Nilsson 2001 [110] suggests several techniques for sampling epiphytes from macrophytes including sonicating, scraping, shaking and chemical treatments. Because there is no standard protocol to study the seagrass microbiome, the different methods used may lead to varying results between studies.

## 5. Conclusions

In conclusion, the microbial communities of the different sample-types differ from one another, but are similar between the two species of seagrasses. Although some of the taxa were present in most of the sample-types, the relative abundance of the taxa differed from species to species as well as from sample-type to sample-type. Some of the most abundant organisms found in the communities of these seagrasses are involved in important biogeochemical cycles that can benefit their seagrass host, including carbon, nitrogen, and sulfur cycles. When comparing our results with other studies, there is strong evidence that a core microbiome exists for seagrasses.

## Figures and Tables

**Table 1:** Location of sampling sites and physico-chemical parameters of the seawater at each location.

Site	Latitude	Longitude	pH	Temperature (°C)	Salinity (ppt)	Conductivity (µS/cm)
Hobie 1	25.743298	−80.173982	8.05	28.7	9.79	19.55
Hobie 2	25.736188	−80.167161	8.28	30.4	9.48	18.95
Hobie 3	25.737209	−80.167979	8.2	31	9.36	18.72

**Table 2:** Total number of Sequence Variants (SV) per seagrass-associated habitats. Three replicates per sample. (T: turtle grass; M: manatee grass; C: control).

Sample Type	Number of SV
Roots T	3373
Roots M	2771
Leaves T	3347
Leaves M	4062
Rhizome T	3597
Sediment C	4650
Sediment T	4033
Sediment M	4033
Seawater	757
All samples	17686

**Table 3:** Comparison of abundant bacteria found in different seagrass studies around the world. Taxa level is based on how the authors ranked their taxa. (p: phylum; c: class; o: order; f: family; nma: not most abundant (most abundant taxa make up more than 1% of the sequences); rfd: removed from data; + present in high relative abundance).

Taxa	Level	Higher Rank	This Study	Cúcio et al. 2016 [77]	Mejia et al. 2016 [86]	Ettinger et al. 2017 [89]	Fahimipour et al. 2017 [90]	Crump et al. 2018 [76]	Bengtsson et al. 2017 [88]
Acidimicrobiia	c	p Actinobacteria	+	+	+		+	+	+
Acidobacteria	p		nma	+					+
Alphaproteobacteria	c	p Proteobacteria	+	+	+	+	+	+	+
Alteromonadales OM60	o/f	c Gammaproteobacteria	+			+			
Anaerolineae	c	p Chloroflexi	+	+					
Bacteroidia	c	p Bacteroidetes	+		+	+		+	
Betaproteobacteria	c	p Proteobacteria	nma		+		+	+	+
Caldithrixae	c	p Bacteroidetes	+	+					
Campylobacterales	o	c Epsilonproteobacteria	nma			+			
Chloroflexi	p		+	+	+				+
Clostridia	c	p Firmicutes	nma	+	+	+		+	+
Cyanobacteria	p		nma (chloroplasts rfd)		+				+
Cytophagia	c	p Bacteroidetes	+	+					+
Deinococcus-Thermus	p								+
Deltaproteobacteria	c		+	+	+	+	+	+	
Desulfobacterales	o	c Deltaproteobacteria	+			+			
Desulfovibrionaceae	f	o Desulfovibrionales, c Deltaproteobacteria	nma				+		
Desulfuromonadaceae	f	o Desulfuromonadales, c Deltaproteobacteria	nma				+		
Epsilonproteobacteria	c	p Proteobacteria	nma	+	+			+	
Flavobacteriia	c	p Bacteroidetes	+		+	+			+
Gammaproteobacteria	c	p Proteobacteria	+	+	+	+	+	+	+
Gemmatimonadetes	p		nma	+					+
Phycisphaerae	c	p Planctomycetes	nma		+				
Plantomycetia	c	p Planctomycetes	+		+		+		+
Plastid	plant	Chloroplasts	+ (rfd)						+
Saprospirae	f	c Sphingobacteria, o Sphingobacteriales	+			+			
Sphingobacteriia	c	p Bacteroidetes	nma		+				+
Spirochaetes	p		+					+	
Verrucomicrobia	p		nma		+				+
WS3	p		nma	+	+				
		Seagrass Species	*T. testudinum* and *S. filiforme*	*Z. marina*, *Z. noltii* and *C. nodosa*	*H. stipulacea*	*Z. marina*	*Z. marina*	*Z. marina* and *Z. japonica*	*Z. marina*
		Location	Key Biscayne, FL	Culatra Island, Portugal	Israel	Bodega bay, California	World wide	Netarts Bay or USA	Hiddensee, Germany

## Supplementary Materials

The following are available online at http://www.mdpi.com/2076-2607/7/1/4/s1, Figure S1: Boxplots of alpha diversity based on phylogenetic diversity (Faith’s PD), Figure S2: Rarefaction curve analysis of the sequences variants of all samples, Figure S3: Venn diagram comparing the most common SVs of the phyllospheres and rhizospheres of both species and the turtle grass rhizomes, Figure S4: Venn diagrams comparing the most common SVs of the seagrass samples from each sampling sites, Hobie 1, Hobie 2 and Hobie 3, Figure S5: Venn diagram comparing the most common SVs of the seawater samples from each sampling site, Hobie 1, Hobie 2 and Hobie 3, Figure S6: Relative abundance of the classes comprising more than 1% of the bacterial sequences in each sample. Taxa are classified to the lowest rank possible according the classifier, Figure S7: Relative abundance of the genera comprising between 0.1% and 1% of the bacterial sequences in each sample, Figure S8: Linear discriminant analysis (LDA) scores of the top 25 most significant genes from the predictive metagenome of the microbial communities in each sample type, Table S1: Pairwise analysis of sample types. (Kruskal Wallis one-way ANOVA test using Faith PD alpha diversity; q-values are adjusted *p*-values with a Benjamin & Hochberg correction), Table S2: Pairwise analysis of seagrass species and the sediment controls. (Kruskal Wallis one-way ANOVA test using Faith PD alpha diversity; q-values are adjusted *p*-values with a Benjamin & Hochberg correction), Table S3: Linear discriminant analysis effect size (LEfSe) of the most significant genes from the predictive metagenome of the microbial communities in each sample type.
