# Supplementary material for: The Microbial Communities of Leaves and Roots Associated with Turtle Grass (Thalassia testudinum) and Manatee Grass (Syringodium filliforme) are Distinct from Seawater and Sediment Communities, but Are Similar between Species and Sampling Sites
Source: Microorganisms. 2018 Dec 26;7(1):4. doi: 10.3390/microorganisms7010004 (PMC6352278; doi:10.3390/microorganisms7010004)
Supplement: Supplementary file 1 [file microorganisms-07-00004-s001.pdf]

microorganisms

MDPI

Supplementary material

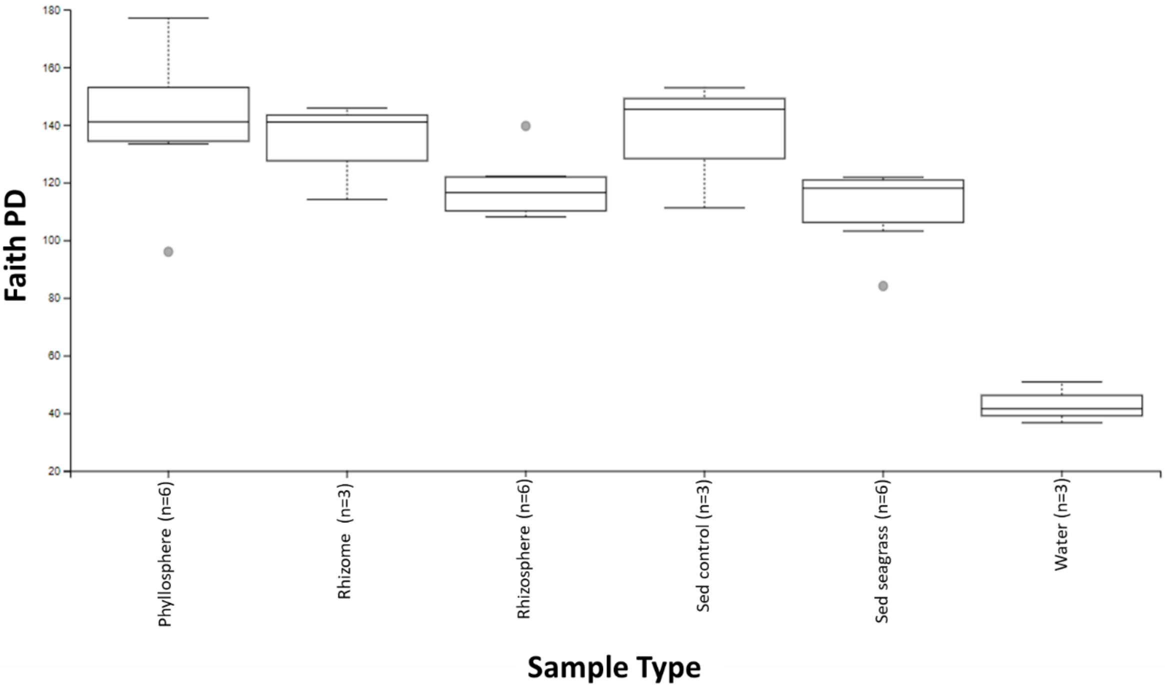

Figure S1. Boxplots of alpha diversity based on phylogenetic diversity (Faith's PD). Table S1 shows associated information.

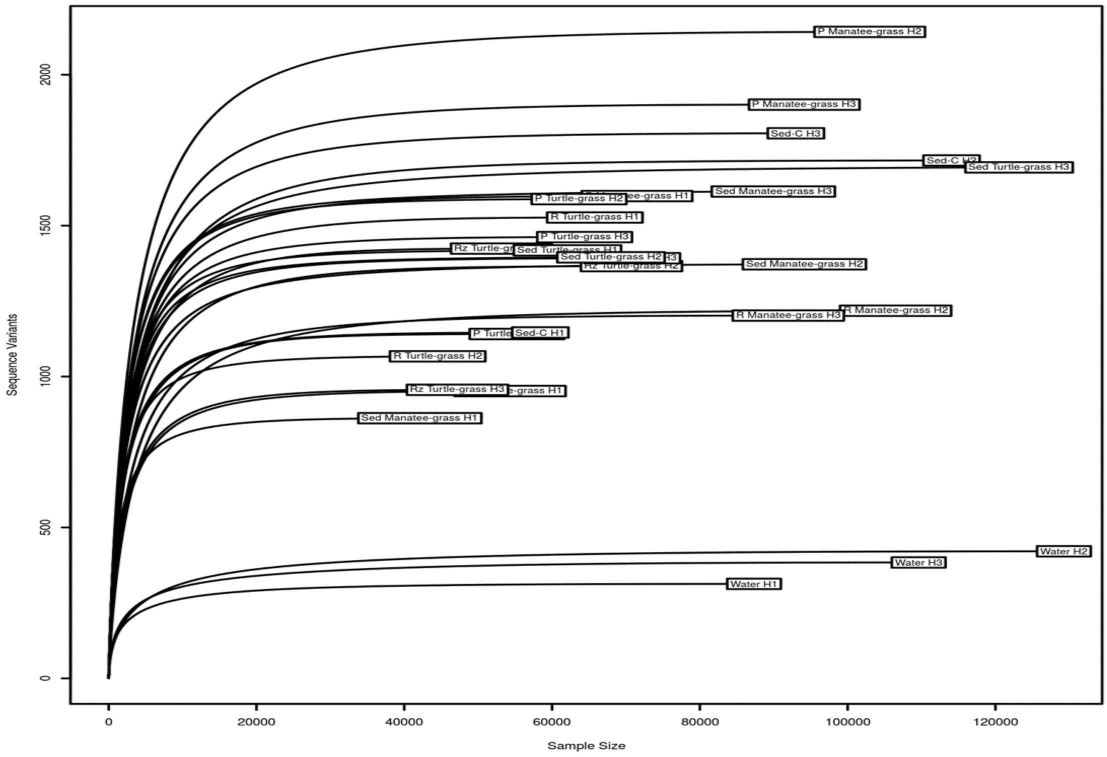

Figure S2. Rarefaction curve analysis of the sequences variants of all samples.

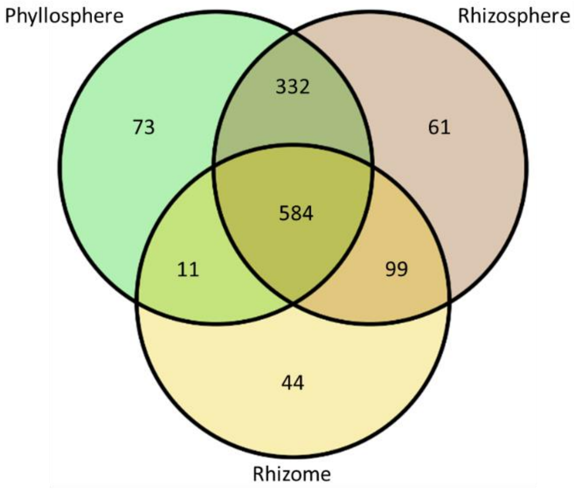

Figure S3. Venn diagram comparing the most common SVs that comprise more than 0.01% of the total SVs unique to or shared among the phyllospheres and rhizospheres of both species and the turtle grass rhizomes.

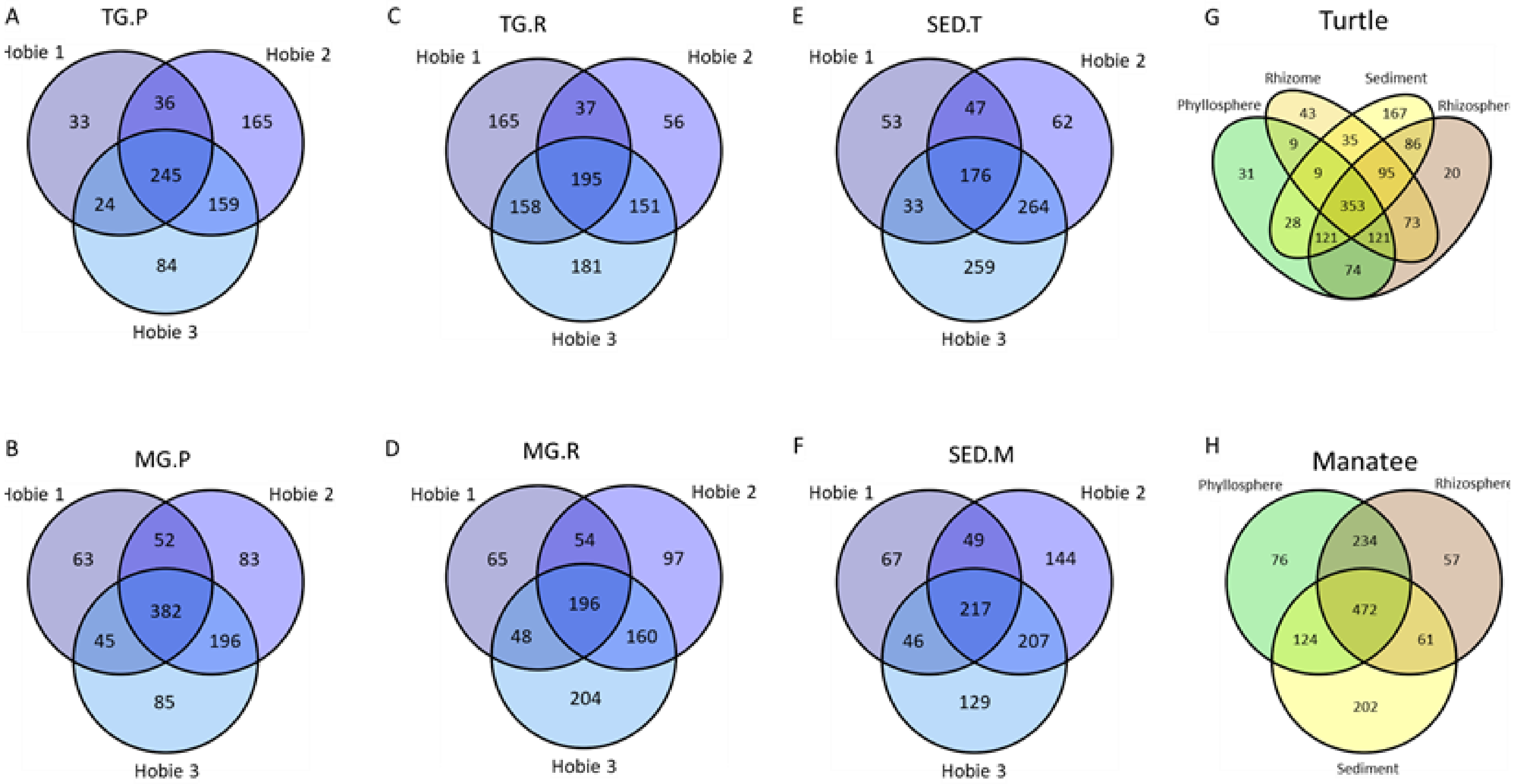

Figure S4. Venn diagrams comparing the most common SVs that comprise more than 0.01% of the total SVs unique to or shared among the three sampling sites, Hobie 1, Hobie 2 and Hobie 3, from (a) turtle grass leaves, (b) manatee grass leaves, (c) turtle grass roots, (d) manatee grass roots, (e) turtle grass sediment, (f) manatee grass sediment; comparison of the common SVs of the different plant segments of (g) turtle grass and (h) manatee grass.

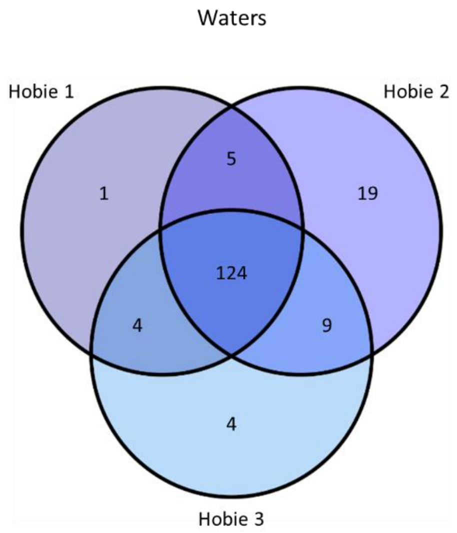

Figure S5. Venn diagram comparing the most common SVs that comprise more than 0.01% of the total SVs unique to or shared among the seawater samples from each sampling site, Hobie 1, Hobie 2 and Hobie 3.

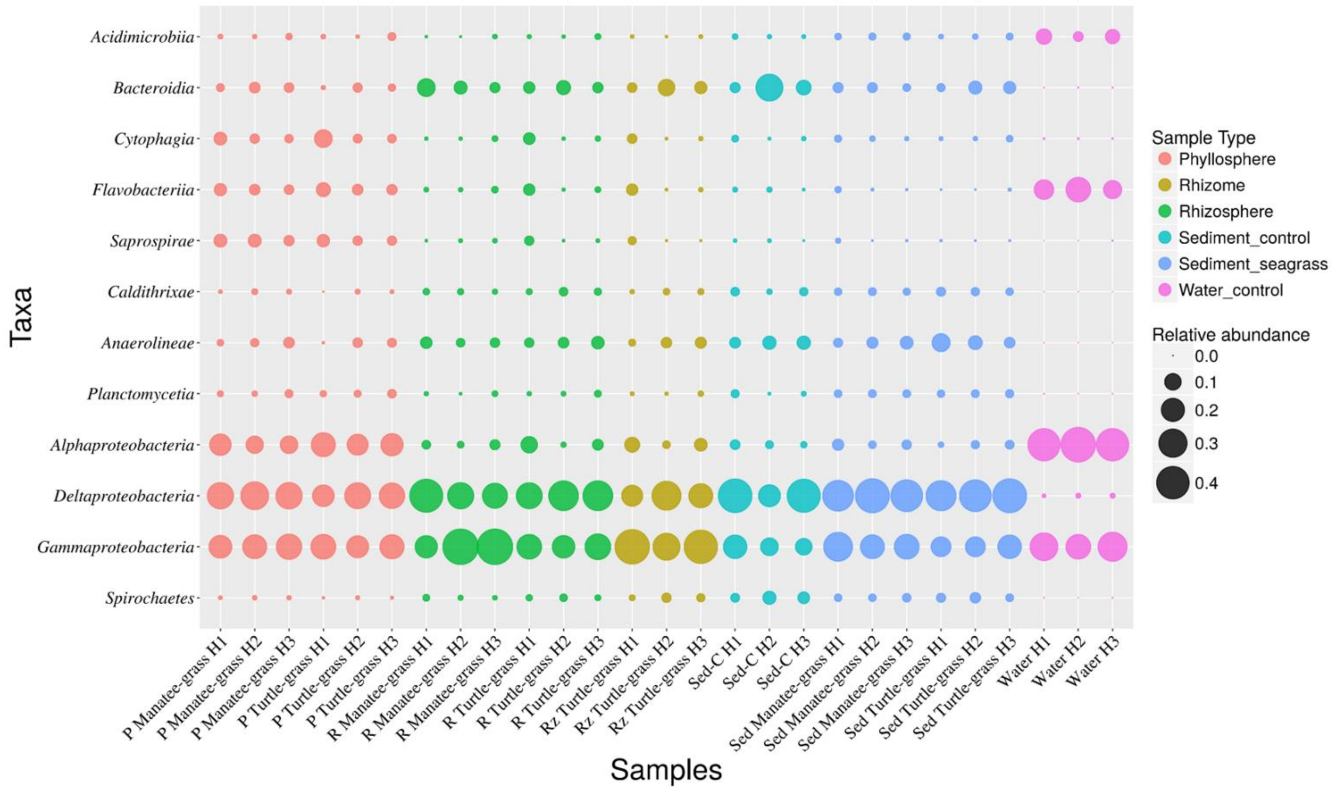

Figure S6. Relative abundance of the classes comprising more than 1% of the bacterial sequences in each sample. Taxa are classified to the lowest rank possible according the classifier. (H1: Hobie 1; H2: Hobie 2; H3: Hobie 3; P: phyllosphere; R: roots; Rz: rhizome; C: control; Sed: sediment)

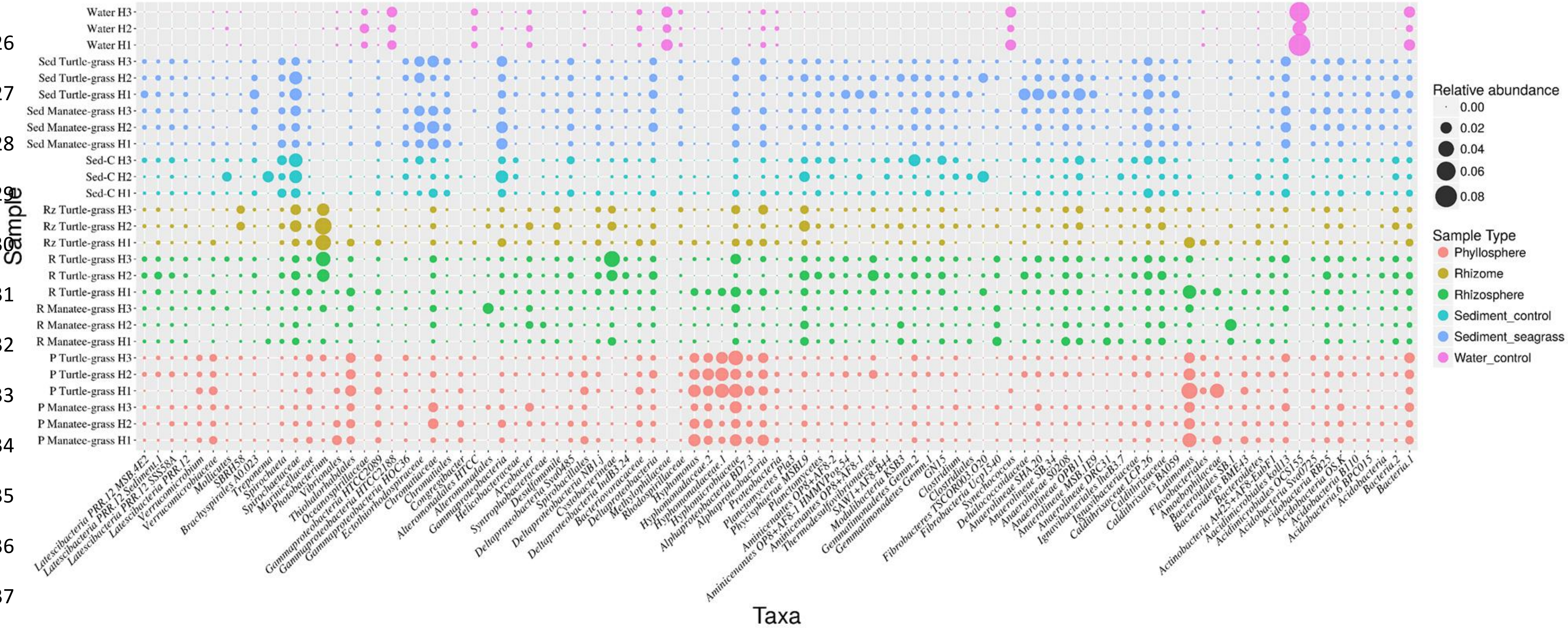

Figure S7. Relative abundance of the genera comprising between 0.1% and 1% of the bacterial sequences in each sample. Taxa are classified to the lowest rank possible according the classifier. (H1: Hobie 1; H2: Hobie 2; H3: Hobie 3; P: phyllosphere; R: roots; Rz: rhizome; C: control; Sed: sediment).

Table S1. Pairwise analysis of sample types. (Kruskal Wallis one-way ANOVA test using Faith PD alpha diversity; q-values are adjusted *p*-values with a Benjamin & Hochberg correction).

Group 1	Group 2	H	*p*-value	q-value
Phyllosphere (n=6)	Water_control (n=3)	5.40	0.02	0.10
Rhizosphere (n=6)	Water_control (n=3)	5.40	0.02	0.10
Sediment_seagrass (n=6)	Water_control (n=3)	5.40	0.02	0.10
Phyllosphere (n=6)	Sediment_seagrass (n=6)	4.33	0.04	0.12
Rhizome (n=3)	Water_control (n=3)	3.86	0.05	0.12
Sediment_control (n=3)	Water_control (n=3)	3.86	0.05	0.12
Phyllosphere (n=6)	Rhizosphere (n=6)	2.56	0.11	0.23
Rhizome (n=3)	Rhizosphere (n=6)	2.40	0.12	0.23
Rhizome (n=3)	Sediment_seagrass (n=6)	1.67	0.20	0.27
Rhizosphere (n=6)	Sediment_control (n=3)	1.67	0.20	0.27
Sediment_control (n=3)	Sediment_seagrass (n=6)	1.67	0.20	0.27
Rhizosphere (n=6)	Sediment_seagrass (n=6)	0.64	0.42	0.53
Phyllosphere (n=6)	Rhizome (n=3)	0.07	0.80	0.89
Rhizome (n=3)	Sediment_control (n=3)	0.05	0.83	0.89
Phyllosphere (n=6)	Sediment_control (n=3)	0.00	1.00	1.00

Table S2. Pairwise analysis of seagrass species and the sediment controls. (Kruskal Wallis one-way ANOVA test using Faith PD alpha diversity; q-values are adjusted *p*-values with a Benjamin & Hochberg correction).

Group 1	Group 2	H	*p*-value	q-value
Turtle-grass (n=12)	Water (n=3)	6.75	0.01	0.04
Manatee-grass (n=9)	Water (n=3)	6.23	0.01	0.04
Sediment-control (n=3)	Water (n=3)	3.86	0.05	0.10
Sediment-control (n=3)	Turtle-grass (n=12)	1.02	0.31	0.47
Manatee-grass (n=9)	Sediment-control (n=3)	0.42	0.52	0.62
Manatee-grass (n=9)	Turtle-grass (n=12)	0.13	0.72	0.72

microorganisms MDPI

1

2 **Table S3**. Linear discriminant analysis effect size (LEfSe) of the most significant genes from the predictive metagenome of the microbial communities in each sample type.

KEGG ID	Function	P-values	Leaves	Roots	Rhizome	Sediment	Sed-control	Water-control	LDA score
**K00845**	glk; glucokinase	0.000179	8.035619	7.087147	6.626892	5.293922	6.077895	**13.428250**	0.705000
**K03293**	amino acid transporter, AAT family	0.000261	4.032242	3.587944	2.892067	2.321494	2.528329	**4.896308**	0.359000
**K08309**	soluble lytic murein transglycosylase	0.000268	10.126992	8.282040	7.727601	6.392645	6.988584	**13.932479**	0.679000
**K02029**	polar amino acid transport system permease protein	0.000273	27.067871	23.448377	20.051279	15.072857	15.227962	**36.912017**	1.080000
**K01620**	threonine aldolase	0.000283	3.829168	3.222452	3.238572	2.618063	2.638476	**11.277951**	0.727000
**K02030**	polar amino acid transport system substrate-binding protein	0.000299	19.309256	17.148610	13.604587	10.791966	11.096586	**25.894535**	0.932000
**K01043**	undetermined	0.000302	2.678694	1.648248	1.849810	1.217936	1.245954	**4.381189**	0.412000
**K00100**	butanol dehydrogenase	0.000368	16.678017	13.816362	10.830087	8.915192	9.597759	**21.165478**	0.853000
**K08988**	putative membrane protein	0.000395	1.333794	1.091106	0.742233	0.593760	0.596058	**1.615858**	0.179000
**K05832**	putative ABC transport system permease protein	0.000396	2.235991	1.561794	1.317041	0.888840	0.970719	**3.902992**	0.399000
**K09788**	2-methylaconitate isomerase	0.000396	0.755371	0.555600	0.579104	1.017602	0.751385	**1.262323**	0.131000
**K04762**	ribosome-associated heat shock protein Hsp15	0.000414	2.310915	2.116422	2.090539	1.657719	1.634073	**3.530218**	0.290000
**K12254**	NAD-dependent aldehyde dehydrogenase	0.000423	0.061807	0.046703	0.048555	**0.203810**	0.128794	0.134089	0.032800
**K05833**	putative ABC transport system ATP-binding protein	0.000431	1.942048	1.371242	1.157186	0.783497	0.850242	**3.361091**	0.360000
**K01286**	D-alanyl-D-alanine carboxypeptidase	0.000443	5.345353	4.177256	4.875712	2.888388	3.071168	**12.998411**	0.782000
**K01989**	putative ABC transport system substrate-binding protein	0.000444	2.713635	2.105066	1.672137	1.299262	1.371952	**4.053225**	0.376000
**K08223**	MFS transporter, FSR family, fosmidomycin resistance protein	0.000448	4.363350	3.724128	2.508803	2.124171	2.773647	**8.659608**	0.630000
**K13954**	alcohol dehydrogenase	0.000463	8.636313	**10.133672**	5.055904	4.592823	5.183233	0.283153	0.773000
**K01468**	hutI, AMDHD1; imidazolonepropionase	0.000465	3.607803	3.002708	2.574716	1.983094	2.276904	**11.299563**	0.753000
**K01958**	pyruvate carboxylase	0.000468	6.439184	4.465530	4.102776	2.713849	3.366694	**17.151255**	0.915000
**K00996**	undecaprenyl-phosphate galactose phosphotransferase	0.000477	1.110619	0.929308	0.823463	0.749728	0.824593	**1.896633**	0.197000

**K03465**	thymidylate synthase	0.000481	**2.813805**	2.442937	1.300913	1.006531	1.121770	0.574566	0.326000
**K07469**	aldehyde oxidoreductase	0.000490	5.524032	**6.362508**	3.105294	2.685889	3.010574	0.021235	0.620000
**K06872**	uncharacterized protein	0.000496	4.338583	3.957716	4.016613	2.796843	3.440344	**7.892967**	0.550000
**K03300**	citrate-Mg2+:H+ or citrate-Ca2+:H+ symporter, CitMHS family	0.000496	0.249924	0.205953	0.173305	0.449186	0.340620	**0.524211**	0.070200
K03535	MFS transporter, ACS family, glucarate transporter	0.000519	0.404577	0.402211	0.466396	**0.656721**	0.584688	0.211186	0.087300
K09972	amino acid transport system ATP-binding protein	0.000520	2.488648	2.142252	1.691467	1.387480	1.510278	**4.723289**	0.426000
K12506	ispDF	0.000535	4.426295	2.879025	2.350607	1.485751	1.446237	**7.703686**	0.616000
K07670	two-component system, OmpR family, response regulator MtrA	0.000538	0.184034	0.235983	0.139221	0.140758	**0.241005**	0.113645	0.026800
K12263	cytochrome c551	0.000540	0.000000	0.000000	0.006443	0.000000	0.000000	**0.221783**	0.045700

microorganisms

MDPI

Figure S8. Linear discriminant analysis (LDA) scores of the top 25 most significant features from the predictive metagenome of the microbial communities in each sample type. Features are described as genes under the KEGG ID column on Table S3.

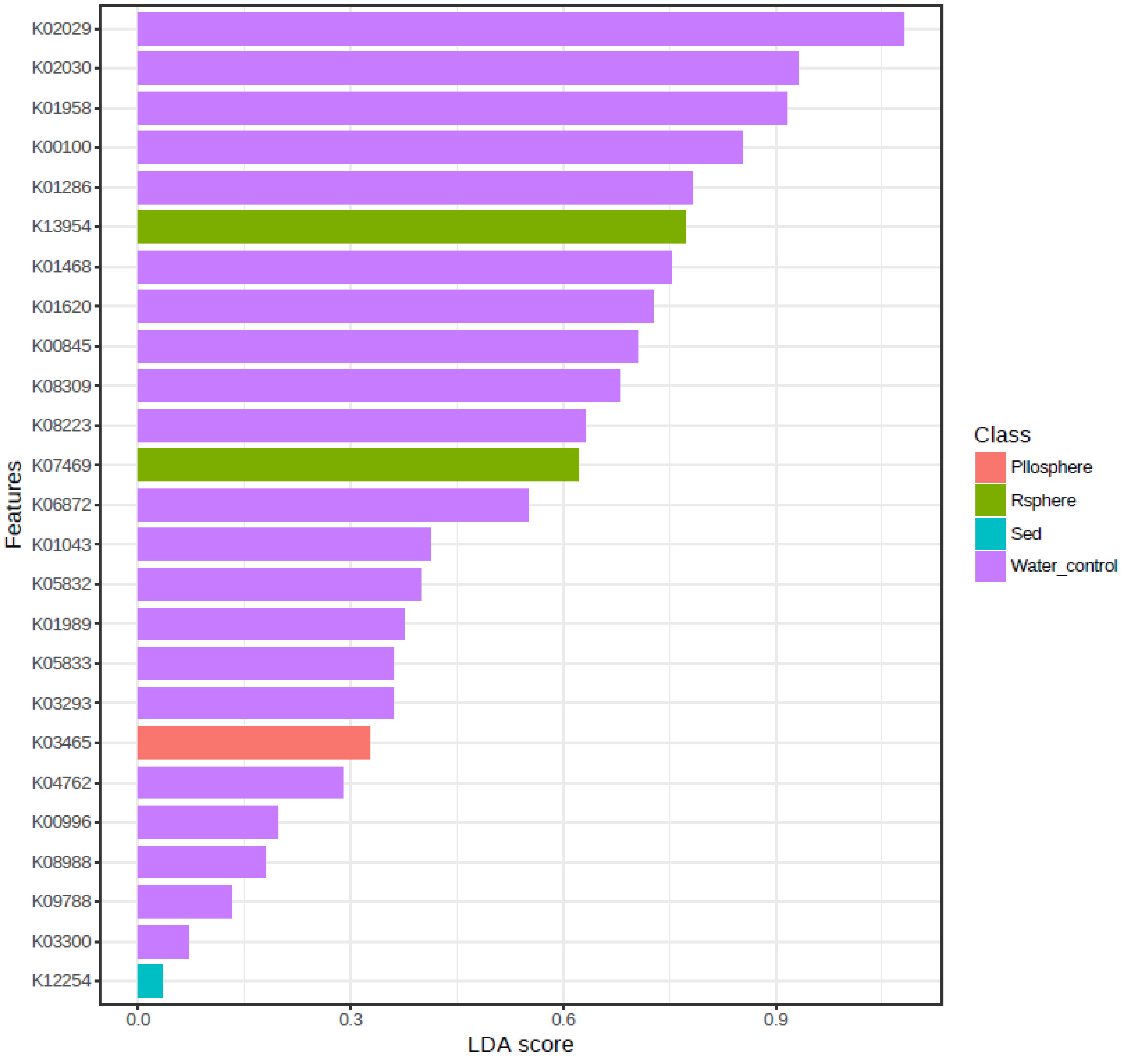

11
